# Applying the Metafounders Approach for Genomic Evaluation in a Multibreed Beef Cattle Population

**DOI:** 10.3389/fgene.2020.556399

**Published:** 2020-12-03

**Authors:** Vinícius Silva Junqueira, Paulo Sávio Lopes, Daniela Lourenco, Fabyano Fonseca e Silva, Fernando Flores Cardoso

**Affiliations:** ^1^Departamento de Zootecnia, Universidade Federal de Viçosa, Viçosa, Brazil; ^2^Breeding Research Department, Bayer Crop Science, Uberlândia, Brazil; ^3^Department of Animal and Dairy Science, University of Georgia, Athens, GA, United States; ^4^Embrapa Pecuária Sul, Bagé, Brazil

**Keywords:** unknown parent group, founder, genomic selection, missing pedigree, breeding values

## Abstract

Pedigree information is incomplete by nature and commonly not well-established because many of the genetic ties are not known *a priori* or can be wrong. The genomic era brought new opportunities to assess relationships between individuals. However, when pedigree and genomic information are used simultaneously, which is the case of single-step genomic BLUP (ssGBLUP), defining the genetic base is still a challenge. One alternative to overcome this challenge is to use metafounders, which are pseudo-individuals that describe the genetic relationship between the base population individuals. The purpose of this study was to evaluate the impact of metafounders on the estimation of breeding values for tick resistance under ssGBLUP for a multibreed population composed by Hereford, Braford, and Zebu animals. Three different scenarios were studied: pedigree-based model (BLUP), ssGBLUP, and ssGBLUP with metafounders (ssGBLUPm). In ssGBLUPm, a total of four different metafounders based on breed of origin (i.e., Hereford, Braford, Zebu, and unknown) were included for the animals with missing parents. The relationship coefficient between metafounders was in average 0.54 (ranging from 0.34 to 0.96) suggesting an overlap between ancestor populations. The estimates of metafounder relationships indicate that Hereford and Zebu breeds have a possible common ancestral relationship. Inbreeding coefficients calculated following the metafounder approach had less negative values, suggesting that ancestral populations were large enough and that gametes inherited from the historical population were not identical. Variance components were estimated based on ssGBLUPm, ssGBLUP, and BLUP, but the values from ssGBLUPm were scaled to provide a fair comparison with estimates from the other two models. In general, additive, residual, and phenotypic variance components in the Hereford population were smaller than in Braford across different models. The addition of genomic information increased heritability for Hereford, possibly because of improved genetic relationships. As expected, genomic models had greater predictive ability, with an additional gain for ssGBLUPm over ssGBLUP. The increase in predictive ability was greater for Herefords. Our results show the potential of using metafounders to increase accuracy of GEBV, and therefore, the rate of genetic gain in beef cattle populations with partial levels of missing pedigree information.

## Introduction

Pedigree information is incomplete by nature and commonly not well-established because many of the genetic ties existent between genealogical information on individuals are not known *a priori* or can be wrong (Junqueira et al., 2017). Nonetheless, pedigrees are usually available for livestock species and have been widely used in genetic evaluations to improve the accuracy of breeding value estimation.

New opportunities to assess relationships between individuals arose in the genomic era. As expected, genomic-based relationships are independent of pedigree information and, therefore, are not affected by missingness or incorrect pedigree recording. Several genomic prediction methods are available in the literature (Meuwissen et al., 2001; VanRaden, 2008; Aguilar et al., 2010; Fernando et al., 2014). Some of the methods (i.e., BayesX, SNP-BLUP, and GBLUP) implicitly assume that pedigree structure is absent (Christensen, 2012), and the extension to several populations, including multiple breeds, is not straightforward because it requires that pedigree and genomic information is compatible (Harris and Johnson, 2010; Misztal et al., 2013). The challenge under genomic approaches is the correct inference of the genetic base population. Usually, the base population for genomic models is assumed to be the available set of genotyped individuals, which is mainly composed of recent animals. In models that combine genomic and pedigree relationships, i.e., ssGBLUP (Aguilar et al., 2010), the compatibility between the pedigree and the genomic base is crucial to avoid bias in GEBV (Vitezica et al., 2011). However, taking care of this compatibility does not solve the issue of limited pedigree recording. Because pedigrees for animal populations only started being recorded recently, the fact that animals could be related before that is ignored.

When multiple breeds are combined in the same evaluation, there is usually no pedigree information between breeds. However, Porto-Neto et al. (2013) and Decker et al. (2014b) showed that cattle populations had common founders. Christensen (2012) provided some insights on how to estimate founder relationships. His suggestions are valid when a single population is assumed *a priori*; however, inference extensions to several founder populations were not exploited. Legarra et al. (2015) reported a metafounder theory to consider relationships within and across founder populations; this theory provided a generalization of unknown parent groups and the developments shown by Christensen (2012). The metafounder concept relies on the definition of pseudo-individuals that add some level of genetic relationship between base individuals in the population (i.e., founders). In this context, we aimed to evaluate the impact of metafounders on the estimation of breeding values for tick resistance under a ssGBLUP model for a multibreed population composed by Hereford, Braford, and Zebu animals.

## Materials and Methods

Approval of Animal care and use committee was not needed because this study used existing datasets historically collected by the animal breeding program. The raw data cannot be made public available because they are property of the Braford and Hereford producers, Embrapa, and GenSys Consultants (i.e., data are commercially sensitive). For research purposes, the data requests should be forwarded along with the research proposal to fernando.cardoso@embrapa.br.

### Phenotype, Genotype, and Pedigree Information

The data used for investigating the inclusion of metafounders in genomic evaluations were provided by *Conexão Delta G* Breeding Program (Rio Grande do Sul, Brazil). Hereford and Braford animals from eight herds had log-transformed tick counts recorded. Braford is a breed resultant of a crossing between Hereford and Zebu (e.g., Nellore, Brahman, Guzerá). A detailed descriptive statistic for the log-transformed tick count is in Table 1. Animals were between 326 and 729 days old at the time of recording. The contemporary groups combined farm, gender, year of birth, management group, and tick count date. Contemporary groups discarded from the dataset had less than five animals and tick counts above or below 3.5 standard deviations from the mean. After editing, 146 contemporary groups remained for further analysis. The phenotypic data included records from 4,363 animals (928 Hereford and 3,425 Braford) raised under extensive conditions, and the pedigree file included 12,755 animals. A total of 35.68% of the animals in pedigree had both parents known, 20.10% of the animals had unknown sire, 0.24% had unknown dam, and 43.98% had both parents unknown (i.e., base animals). Among all phenotyped individuals, 2,188 (525 Hereford and 1,663 Braford) had three subsequent tick counts, 1,934 (391 Hereford and 1,543 Braford) had two counts, and 241 (12 Hereford and 229 Braford) had only one count. Therefore, a total of 10,673 tick counts were recorded on 2,369 Herefords, and on 8,304 Brafords that had a maximum of 3/4 of Zebu proportion. The Zebu breed proportion, heterozygosity, and recombination loss effects were calculated as proposed by Cardoso and Tempelman (2004) and included as linear covariates in the model.

**TABLE 1:** Descriptive statistics of the log-transformed tick count records for Hereford and Braford.

**Descriptive statistics**^*a*^	**Breed**	
	**Hereford**	**Braford**
N	2,369	8,304
Minimum	0.0004	0.0004
Q25	1.18	1.11
Mean	1.45	1.33
Median	1.46	1.38
Q75	1.74	1.60
Maximum	2.73	2.72
SD	0.47	0.43

*^*a*^N, number of observations; Q25, quantile 25%; Q75, quantile 75%; SD, standard deviation.*

In total 130 sires were genotyped with a high-density SNP panel (BovineHD—Illumina bead chip with 777,962 SNPs), whereas the BovineSNP50 Illumina panel (54,609 SNPs) was used to genotype 3,591 animals. A total of 41,045 overlapping SNPs were selected for quality control. The quality control criteria adopted for SNP exclusion were the Hardy–Weinberg equilibrium chi-square test (*p* = 10^–7^), genotype call rate (CR) (<98%), minor allele frequency (MAF) (<3%), near-perfect collinearity with other SNPs (*r* > 0.98), and SNPs in the same physical position. The criteria adopted to reject samples were *CR* < 90%, heterozygosity deviation above three standard deviations, gender identification errors, and identical genotypes between different individuals (more than 99.5% of similarity for all markers). After quality control, a total of 3,591 samples (666 Braford and 2,862 Hereford) and 39,550 markers were retained for further analysis. Aiming to build a complete 39,550 marker panel, missing genotypes (0.89% of all genotypes) were imputed across breeds according to the sliding window method using FImpute (Sargolzaei et al., 2011).

### Metafounder Relationships

The metafounder relationship used in this study was derived from the methodology proposed by Legarra et al. (2015). In summary, their approach is a general framework that considers each ancestral population containing a finite-sized pool of gametes. Conceptually, that assumption contrasts with the classical population genetics supposition and suggests that several ancestral populations might be genetically related, and therefore, connected. In the aforementioned paper, the authors presented modifications to the pedigree-based relationship matrix for populations under different structures (i.e., single and multiple base populations). The concept of metafounder relies on the definition of pseudo-individuals to add some level of within and/or across genetic relationships between base (i.e., founder or ancestral, γ = 1/*Ne*) individuals in the population. It is assumed that every individual from any population might have some degree of known or unknown relationship due to a common ancestor. From the perspective of founder individuals, their relationship can be derived by the use of metafounders, constructing a modified pedigree relationship matrix, **A**(Γ). The Γ matrix contains the relationship between metafounders (composed by at least one γ), and its simplest form is exhibited when the ancestral population is composed of only one breed, indicating that Γ is a scalar. In cases where the founder population is composed of several populations and eventually, with crossbred animals, it is possible to build an extended and more complex Γ. The latter is exactly the case of the population used in this study, which is composed of Hereford and Braford (an admixture between Hereford and Zebu) animals.

A total of four metafounders were defined based on breed of origin, with one metafounder assigned to Hereford, another one for Braford, and a third one for Zebu. The fourth metafounder was assigned to the remaining base animals with an unknown breed of origin. The description of each metafounder group is in Table 2. Recursive computations of **A**(Γ) followed usual rules (Emik and Terrill, 1949; Karigl, 1981; Aguilar and Misztal, 2008). The only required modification to include metafounders is the assumption of γ as the self-relationship for founders. Note that self-relationship for base animals is traditionally assumed to be zero due to lack of historical pedigree information. The Γ matrix, which is composed by within- and across-founder relationships, was estimated using SNP markers under a generalized least square (GLS) approach (Garcia-Baccino et al., 2017). In our study, Γ was a 4 × 4 (co)variance matrix between means across markers and breeds. Below is a description of the GLS linear model fitted in this study where the breeding values are split into within- and across-breed components:

**TABLE 2:** Number of males and females included in pedigree in each metafounder constructed based on breed of origin and within (diagonal) and across (off-diagonals) gamma values (Γ) estimated using generalized least square.

			**Γ**			
			
**Metafounders**	**Males**	**Females**	**Hereford**	**Bradford**	**Zebu**	**Unknown**
Hereford	1,991	1,032	0.61	0.46	0.34	0.49
Braford	3,932	2,431		0.53	0.57	0.50
Zebu	34	34	Symm		0.96	0.52
Unknown	1,228	1,084				0.51

$${\text{m}}_{i}=\text{Q}\,\mu_{i}+\sum_{b}{\text{W}}^{b}\,{\text{u}}_{i}^{b}+\sum_{b,b^{\prime },b>b^{\prime }}{\text{W}}^{b,b^{\prime }}\,{\text{u}}_{i}^{b,b^{\prime }}+{\text{e}}_{i},$$

where **m**_*i*_ is a vector of gene contents in the form [0, 1, 2] from locus *i*, **Q**_*k*,*b*_ is a matrix, the rows of which sum to 1, and contains the fraction of ancestry *b* in individuals *k*, μ_*i*_ is a vector for the average of each population, **W**^*b*^ is an incidence matrix relating individuals from *b* group in the pedigree to observed genotypes, with partial relationship matrices for vectors ${\text{u}}_{i}^{b}∼N\,\left(0,{\text{A}}^{b}\,\left(2\,p_{i}\,\left(1-p_{i}\right)\right)\right)$ and ${\text{u}}_{i}^{b,b^{\prime }}∼N\,\left(0,{\text{A}}^{b,b^{\prime }}\,\left(2\,p_{i}\,\left(1-p_{i}\right)\right)\right)$, and **A**^*b*(*b*,*b*′)^ the matrix of pedigree-based relationships among individuals in population *b*. The residual term can be defined as $\text{e}∼N\,\left(0,\sigma_{\varepsilon }^{2}\right)$. The BLUE of μ_*i*_ can be obtained and then the variance and covariance between means for markers within and across populations $\left(\hat{\Sigma }\right)$ are estimated. Finally, Γ was estimated as $\Gamma =2\,\hat{\Sigma }=2\,\left(\begin{array}{cccc} \sigma_{\mu_{B}}^{2} & \sigma_{\mu_{B}\,\mu_{H}} & \sigma_{\mu_{B}\,\mu_{Z}} & \sigma_{\mu_{B}\,\mu_{u}}\\ & \sigma_{\mu_{H}}^{2} & \sigma_{\mu_{H}\,\mu_{Z}} & \sigma_{\mu_{H}\,\mu_{U}}\\ & & \sigma_{\mu_{Z}}^{2} & \sigma_{\mu_{Z}\,\mu_{U}}\\ s\,y\,m & & & \sigma_{\mu_{U}}^{2} \end{array}\right)$, where $\sigma_{b}^{2}$ and σ_*μ_b^ μ_b’*_ are the variance and covariance parameters for each Hereford (H), Braford (B), Zebu (Z), and unknown breed of origin (U).

### Statistical Models

Three different models were tested in this study, aiming to evaluate the gain in prediction accuracy due to the inclusion of metafounders in genetic evaluations. The first model contained only relationships based on pedigree information (BLUP); the second model was the single-step genomic BLUP (ssGBLUP), which combines pedigree and genomic information; the third model was the ssGBLUP with metafounders (ssGBLUPm). No restrictions were imposed on the approach to avoid or minimize inbreeding, and because of that, a total of 130 inbred individuals out of 72,755 were defined by non-zero inbreeding coefficient. The average inbreeding coefficient from inbred animals was 5.73%, with a maximum of 25%, and 0.06% for all 72,755 animals.

To reduce the computational time for variance components estimation in average information REML (AIREML), the starting values were estimated through pedigree-based model via Gibbs sampling algorithm implemented in GIBBS2F90 (Misztal et al., 2002). This software implements a Bayesian method using Gibbs sampler via the Markov Chain Monte Carlo (MCMC) algorithm. Thus, a Bayesian bivariate pedigree-based repeatability model for tick count was defined as following data distribution:

$${\text{y}}_{i\,j\,k\,l}|\beta ,\gamma ,c,a,d,R∼N\,\left({\text{x}}_{1\,j\,k}\,\beta +{\text{x}}_{2\,j\,k}\,\omega +{\text{x}}_{3\,j\,k}\,\text{c}+{\text{z}}_{k}\,\text{a}+{\text{z}}_{k}\,\text{d},\sigma_{{\text{e}}_{k}}^{2}\right)$$

where **y**_*ijkl*_ is the *l*th log-transformed phenotypic record for breed *k* (1 = Hereford, 2 = Braford) in the *j*th animal, from the *i*th contemporary group; **β** is a vector of systematic effects; ω = [ω_A*k*_ω_D*kk*′_ω_AA*kk*′_] is a vector of Zebu breed proportion, heterozygosity, and recombination loss effects, represented respectively by ω_*A_k*_, ω_*D_kk’*_, and ω_*AA_kk’*_. Additionally, $\text{c}|\text{C}∼N\,\left(0,\left[\begin{array}{c} \begin{array}{cc} \sigma_{{\text{c}}_{1}}^{2} & \sigma_{{\text{c}}_{12}}\\ \sigma_{{\text{c}}_{21}} & \sigma_{{\text{c}}_{2}}^{2} \end{array} \end{array}\right]⊗\text{I}\right)$ is a vector of random contemporary group effects; $\text{a}|{\text{G}}_{o},A∼N\,\left(0,\left[\begin{array}{c} \begin{array}{cc} \sigma_{{\text{a}}_{1}}^{2} & \sigma_{{\text{a}}_{12}}\\ \sigma_{{\text{a}}_{21}} & \sigma_{{\text{a}}_{2}}^{2} \end{array} \end{array}\right]⊗\text{A}\right)$ is a vector of random direct additive genetic effects, where **G**_**o**_ is the additive genetic (co)variance matrix and **A** is the numerator relationship matrix; $\text{d}|\text{D}∼N\,\left(0,\left[\begin{array}{c} \begin{array}{cc} \sigma_{{\text{d}}_{1}}^{2} & \sigma_{{\text{d}}_{12}}\\ \sigma_{{\text{d}}_{21}} & \sigma_{{\text{d}}_{2}}^{2} \end{array} \end{array}\right]⊗\text{I}\right)$ is a vector of random permanent environmental effects. Furthermore, **x**_1*jk*_, **x**_2*jk*_, **x**_3*jk*_ are known vectors, and *z*_*k*_ is an incidence matrix where the elements of *x*_*2jk*_ in column order are follows: (1) *f*_*k*_, defined as the proportion of alleles from the *k*^*th*^ breed and corresponding to ω_*A_b*_; (2) *f*_*kk’*_ being the probability that a randomly chosen locus from an individual *j*, one allele is derived from breed *k* and the other allele is derived from breed *k’*, associated with ω_*D_bb’*_; and (3) $2\,f_{k}\,f_{k^{\prime }}$ corresponding to ω_AA*bb*′_ (Cardoso and Tempelman, 2004). Finally, $\sigma_{{\text{e}}_{k}}^{2}$ is the residual variance for the *k*th trait. Inverted Wishart prior densities are specified for the covariance components as follows: **C**|Σ_**C**_,*n*∼*IW*(Σ_**C**_,*n*), **G**|Σ_**G**_,*n*∼*IW*(Σ_**G**,*n*_), **D**|Σ_**D**_,*n*∼*IW*(Σ_**D**_,*n*), **R**|Σ_**R**_,*n*∼*IW*(Σ_**R**_,*n*), where *Σ*_*q*_ is the respective scale matrix for each q effect and degrees of belief parameter given by n. All effects were fitted using degree of belief equals 1 and Σ_q_→∞ aiming to fit a flat distribution.

A total of 100,000 iterations were generated, with the first 30,000 discarded as burn-in, and 1 every 10th sample was stored for posterior analysis. Posterior means were then used as starting values in AIREMLF90 (Misztal et al., 2002) using the YAMS package for efficient sparse computations (Masuda et al., 2014). AIREMLF90 calculates REML (co)variance estimates with the Average-Information algorithm, which uses a second derivative REML algorithm.

A two-trait repeatability animal model was used to estimate breeding values. The model can be seen as an incomplete version of the development proposed by Wei and Van der Werf (1994) because records from one of the purebreds (Zebu animals) were not available. Notations hereafter follow Wei and Van der Werf (1994). The model can be defined as:

$$\left[\begin{array}{c} {\text{y}}_{\text{H}}\\ {\text{y}}_{\text{B}} \end{array}\right]=$$

$$\left[\begin{array}{cc} {\text{X}}_{\text{H}} & 0\\ 0 & {\text{X}}_{\text{B}} \end{array}\right]\,\left[\begin{array}{c} \beta_{\text{H}}\\ \beta_{\text{B}} \end{array}\right]+\left[\begin{array}{cc} {\text{Z}}_{\text{H}} & 0\\ 0 & {\text{Z}}_{\text{B}} \end{array}\right]\,\left[\begin{array}{c} {\text{a}}_{\text{H}}\\ {\text{a}}_{\text{B}} \end{array}\right]+\left[\begin{array}{cc} {\text{Z}}_{\text{H}} & 0\\ 0 & {\text{Z}}_{\text{B}} \end{array}\right]\,\left[\begin{array}{c} {\text{d}}_{\text{H}}\\ {\text{d}}_{\text{B}} \end{array}\right]+\left[\begin{array}{c} {\text{e}}_{\text{H}}\\ {\text{e}}_{\text{B}} \end{array}\right]$$

where **y**_i_ is the vector of log-transformed tick counts in the *ith* breed – Hereford (H) and Braford (B); **X**_i_, and **Z**_i_, are incidence matrices that relate phenotypes to its respective fixed, direct additive, and permanent environmental effect levels, respectively. The vector of fixed effect (β_i_) was composed by an overall mean and contemporary groups as cross-classified variables; zebu breed proportion, heterozygosity, recombination loss, and linear and quadratic effects of age at tick counting were considered as covariables. The vector of permanent environmental effect was defined as $\text{d}∼N\,\left(0,\left[\begin{array}{cc} \sigma_{{\text{d}}_{\text{H}}}^{2} & \sigma_{{\text{d}}_{\text{HB}}}\\ \sigma_{\text{BH}} & \sigma_{{\text{d}}_{\text{B}}}^{2} \end{array}\right]⊗\text{I}\right)$; and the residual vector as $\text{e}∼N\,\left(0,\left[\begin{array}{cc} \sigma_{{\text{e}}_{\text{H}}}^{2} & 0\\ 0 & \sigma_{{\text{e}}_{\text{B}}}^{2} \end{array}\right]⊗\text{I}\right)$ Moreover, the vector of direct additive effects for BLUP was defined as $\left[\begin{array}{c} {\text{a}}_{\text{H}}\\ {\text{a}}_{\text{B}} \end{array}\right]∼N\,\left(0,\left[\begin{array}{cc} \sigma_{\text{H}}^{2} & \sigma_{\text{HB}}\\ \sigma_{\text{BH}} & \sigma_{\text{B}}^{2} \end{array}\right]⊗\text{A}\right)$; where $\sigma_{H}^{2}$ and $\sigma_{B}^{2}$ are the additive variances for the Hereford and Braford traits, respectively, and σ_*HB*_ is the additive covariance between breeds.

For ssGBLUP and ssGBLUPm, the **A** matrix was replaced by **H** and **H(**Γ), respectively, where **H** is the realized relationship matrix and Γ is a matrix of relationships among metafounders. The **H^–1^** can be defined as following:

$${\text{H}}^{-1}={\text{A}}^{-1}+\left[\begin{array}{cc} 0 & 0\\ 0 & {\left(0.95\,\text{G}+0.05\,{\text{A}}_{22}\right)}^{-1}-{\text{A}}_{22}^{-1} \end{array}\right]$$

where ${\text{A}}_{22}^{-1}$ is the inverse of the pedigree relationships for genotyped animals. The inverse of the realized relationship matrix with metafounder, **H(Γ)^–1^**, was also constructed using the same approach, however, the pedigree-based relationship matrix was constructed as **A**(Γ) instead of **A**; likewise, **A**_22_ was replaced by **A**_22_(Γ). The genomic relationship matrix (**G**), was constructed as:

$$\text{G}=\frac{\left(\text{M}-\text{P}\right)\,{\left(\text{M}-\text{P}\right)}^{{}^{\prime }}}{2\,\sum_{\text{j}=1}^{\text{s}}p_{j}\,\left(1-p_{j}\right)},$$

where **M** is the matrix of SNP genotypes for each animal, **P** is a matrix of two times the frequency of the second allele *p* at locus *j* (*p*_*j*_), and *s* is the number of SNP markers. The denominator is a scaling factor for **G**. Under ssGBLUP, **G** was constructed using realized allele frequencies in the genotyped data, whereas 0.5 allele frequency was used for all loci in ssGBLUPm. VanRaden (2008) suggests the use of allele frequencies from base animals (i.e., unselected population) to create the genomic matrix. However, SNP markers are not available for base animals and approximations needs to be used. In those circumstances, allele frequencies from current population are used to build genomic matrix and scaling diagonal and off-diagonal elements of **G** are required to ensure **A**, **A**_22_, and **G** compatibility in single-step approach (Chen et al., 2011; Forni et al., 2011). The use of 0.5 allele frequency in ssGBLUPm refers to the population with maximum heterozygosity. All **Γ** computations were performed using a new software (GAMMAF90) being developed by BLUPF90 group^1^. The software was written in Fortran 95 and it is integrated in the new BLUPF90 software.

### Scenarios

The BLUP model was fitted using the regular relationship matrix constructed based on Henderson (1976) rules. The relationship matrix used in ssGBLUP and ssGBLUPm was described in the previous section.

To compare the estimated variance components and genetic parameters between models, ssGBLUPm parameters needed to be adjusted corresponding to (co)variances among the unrelated breeds (scaled) (Legarra et al., 2015). More specifically, the scaled genetic variances for Hereford (Braford) were $\sigma_{{\text{a}}_{\text{H}\,\left(\text{B}\right)}}^{2}\,\left(1-\gamma_{\text{H}\,\left(\text{B}\right)}\,/\,2\right)$; the scaled genetic covariance for crossbred performance was σ^aHB^(1−γ_HB_/2). Note the γ_H_ and γ_B_ represents the metafounder genetic relationship within Hereford and Braford, respectively, and γ_HB_ represents the across metafounder genetic relationship between Hereford and Braford. Heritabilities were calculated using these scaled (co)variance components. The genetic correlation between Hereford and Braford was calculated as $r_{a}=\frac{\sigma_{{\text{a}}_{\text{HB}}}\left(1-\gamma_{HB}/2\right)}{\sqrt{\sigma_{{\text{a}}_{\text{H}}}^{2}\left(1-\gamma H/2\right)\sigma_{{\text{a}}_{\text{B}}}^{2}\left(1-\gamma_{B}/2\right)}}$. Finally, repeatability for Hereford and Braford was calculated as $r_{H}=\frac{\sigma_{{\text{a}}_{\text{H}}}^{2}\left(1-\gamma H/2\right)+\sigma_{{\text{d}}_{\text{H}}}^{2}}{\sigma_{{\text{p}}_{\text{H}}}^{2}}$ and $r_{B}=\frac{\sigma_{{\text{a}}_{\text{B}}}^{2}\left(1-\gamma_{B}/2\right)+\sigma_{{\text{d}}_{\text{B}}}^{2}}{\sigma_{{\text{p}}_{\text{B}}}^{2}}$, respectively. The same formulas were used to compute heritability, genetic correlation, and repeatability for BLUP and ssGBLUP models, using the (co)variances estimated by AIREML.

### Within-Breed Predictive Ability

In this study, the within-breed predictive ability was used to measure the model ability to predict unknown phenotypes. For that, we used a forward validation approach. The selection of animals to compose the validation set in the forward validation was based on year of birth. Therefore, training animals were born from 2008 to 2010, and validation animals were born in 2011. A total of 198 and 766 animals were part of validation sets for Hereford and Braford, respectively.

The predictive ability was defined as the correlation between phenotypes adjusted for fixed effects (${\hat{\text{y}}}_{\text{i}}^{*}={\text{y}}_{\text{i}}-{\text{X}}_{\text{i}}\,\beta_{\text{i}}$) from a model using all data where ${\hat{\text{y}}}_{\text{i}}^{*}$ is the adjusted phenotype for animals in the *i*^*th*^ breed (Hereford and Braford) and fixed effects as defined in AIREML model. The predictive ability for Hereford was calculated as cor (${\hat{\text{y}}}_{\text{H}}^{*},{\hat{\text{a}}}_{\text{H}})$ using information only for the validation animals. Similarly, the predictive ability for Braford was computed as cor $\left({\hat{y}}_{B}^{*},{\hat{a}}_{B}\right)$. Standard error for the predictive ability was generated from 5,000 non-parametric bootstrapping replicates. All computation was implemented using boot function from boot R package (Canty, 2002; Team, 2013). Regression of phenotypes adjusted for fixed effects on (G)EBVs for Hereford and Braford was used as a measure of the inflation (bias) of the prediction method, where a regression coefficient of one denotes no bias.

## Results and Discussion

### Metafounder Relationship and Inbreeding

A total of four metafounders were included in the ssGBLUPm model. Three metafounders were defined based on breed of origin (Hereford, Braford, and Zebu) and the last metafounder was assigned to the remaining base animals with unknown breed of origin. Table 2 shows the number of males and females included in each metafounder group.

Self- and across- relationships (Γ) between Hereford, Braford, and Zebu breeds estimated by generalized least squares are also shown in Table 2. As previously defined by Legarra et al. (2015), $\hat{\gamma }$ can be seen as self-relationships. The relationship coefficient between metafounders was greater than zero, suggesting a degree of overlap between ancestor populations. The estimates of metafounder relationships indicate that the Hereford and Zebu populations in our study might have some ancestors in common. However, as previously stated, there is no genomic information for Zebu animals in this study; in fact, only a fraction of all zebu descendants was used for computations. Thus, the population under study is a special case of the metafounder theory (Legarra et al., 2015) where records from one of the pure breeds is unknown, but genomic information for crossbreds is available. Moreover, the SNP panel used in this analysis is a blend of different SNP-chips where the missing genotypes were imputed. Our intention was not to draw any assumptions on how Hereford and Zebu breeds have shared a certain portion of the alleles over generations. For that purpose, there are other approaches already published in the literature (Alexander and Lange, 2011; Decker et al., 2014a).

**FIGURE 1:**
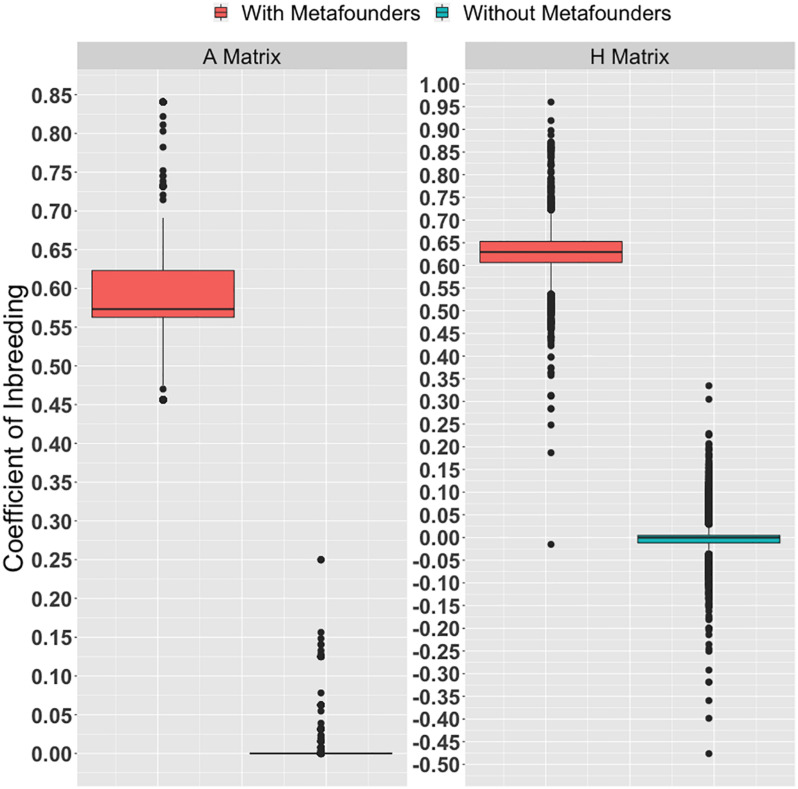
Inbreeding estimates obtained from the diagonal elements of the pedigree **(A)** and the realized **(H)** relationship matrices (with and without metafounders).

The inbreeding coefficients calculated based on pedigree and genomic information (with and without metafounders) are shown in Figure 1. A detailed description of inbreeding coefficients within breed compositions (i.e., Zebu, Hereford, and Braford) are available in Supplementary Figure S1. Many individuals used in this study had missing pedigree information. Due to the lack of information, almost all the diagonal elements in **A** without metafourders are equal zero. Because of the inclusion of metafounders, an upward shift was observed in the inbreeding coefficients calculated based on **A** and **H**. Additionally, a few negative inbreeding coefficients were observed. This result suggests that parents were less related than the average in the base population (assuming allele frequencies of 0.5). The classical quantitative genetics theory postulates that inbreeding for individuals with known parents is a function of parent’s relationships. Founder individuals are typically assumed to be drawn from a large, unrelated, ancestral population mated at random. Consequently, inbreeding coefficients for founder animals are usually defined as zero due to the lack of information. A different condition arises under the metafounder theory where base animals are assumed to be related due to a common ancestral population. In this case, the probability that identical gametes are shared between individuals may increase; thus, inbreeding coefficients are upward shifted (Figure 1).

Additional information about the diagonal and off-diagonal elements of all required matrices to create **H** and **H**(Γ) matrices are available in Table 3. As previously stated, the inclusion of metafounders in the numerator relationship matrix and the assumption of allele frequency equals 0.5 causes an upward shift on **A**_22_ and **G**.

**TABLE 3:** Descriptive statistics for diagonal and off-diagonal elements of genomic matrices required under genomic evaluations.

	**Parameter**			
**Matrix**^*a*^	**Mean**	**Minimum**	**Maximum**	**Variance**

**Diagonal**				
**A**_22_	1.001	1.000	1.250	0.000
**A**_2__2_ (**Γ**)	1.258	1.200	1.447	0.000
**G**	1.001	0.838	1.204	0.002
**G** (**Γ**)	1.289	1.185	1.407	0.001

**Off-diagonal**				

**A**_22_	0.002	0.000	0.750	0.000
**A**_2__2_ (**Γ**)	0.504	0.399	1.079	0.001
**G**	0.002	-0.228	0.678	0.002
**G** (**Γ**)	0.558	0.380	1.051	0.002

*^*a*^***A**_22_, *numerator relationship matrix of genotyped animals;***G**, *genomic matrix;******Γ*****, *matrices using metafounder information.*

### Variance Components, Heritability, and Genetic Correlations

Variance components, heritability, and genetic correlations are available in Table 4. As previously described, variance components from the metafounder model were scaled to provide a fair comparison with BLUP and ssGBLUP models. Across different models, it can be seen that additive genetic, residual, and phenotypic variances estimated based on the Hereford data were smaller than those based on Braford. Permanent environmental variances were similar across models.

**TABLE 4:** Description of variance components, heritability, and genetic correlation estimates (with respect standard-errors) for Hereford and Braford using multibreed pedigree and genomic information.

**Parameters**^*a*^	**Model**^*b*^					
	**BLUP**		**ssGBLUP**		**ssGBLUPm**	
	**Hereford**	**Braford**	**Hereford**	**Braford**	**Hereford**	**Braford**
$\sigma_{\text{a}}^{2}$	0.003 (0.000)	0.027 (0.002)	0.009 (0.004)	0.018 (0.003)	0.013 (0.001)	0.018 (0.002)
$\sigma_{\text{d}}^{2}$	0.018 (0.002)	0.006 (0.001)	0.013 (0.004)	0.013 (0.002)	0.009 (0.001)	0.013 (0.001)
$\sigma_{\text{e}}^{2}$	0.060 (0.000)	0.074 (0.001)	0.060 (0.002)	0.074 (0.002)	0.060 (0.002)	0.074 (0.001)
$\sigma_{\text{p}}^{2}$	0.081 (0.004)	0.106 (0.004)	0.082 (0.011)	0.105 (0.006)	0.082 (0.004)	0.105 (0.004)
*h*^2^	0.040 (0.003)	0.250 (0.003)	0.110 (0.044)	0.170 (0.015)	0.160 (0.005)	0.180 (0.007)
*r*	0.260 (0.013)	0.310 (0.012)	0.260 (0.080)	0.300 (0.030)	0.270 (0.008)	0.300 (0.010)
*r*_*a*_	0.670 (0.022)		0.450 (0.015)		0.410 (0.017)	

*^*a*^*$\sigma_{\text{a}}^{2}$, *additive genetic variance;*$\sigma_{\text{d}}^{2}$, *permanent environment variance;*$\sigma_{\text{e}}^{2}$, *residual variance;*$\sigma_{\text{p}}^{2}$, *phenotypic variance; h*^2^, *additive heritability; r*, *repeatability; r*_*a*_, *genetic correlation. ^*b*^BLUP, pedigree-based BLUP; ssGBLUP, single-step genomic BLUP; ssGBLUPm, ssGBLUP with metafounders. Variance components under ssGBLUPm were scaled following the material and methods description.*

In general, variance components and heritabilities were not considerably different between the genomic models. The most remarkable difference is seen in the heritability estimates on Hereford breed, where the inclusion of metafounders led to an increase of heritability. On the other hand, the inclusion of genomic information resulted in smaller heritability estimates on Braford breed. Both conditions can be attributed to improvements on additive genetic relationships, and consequently, on permanent environment effects estimation. The heritability shift observed between non-genomic and genomic models suggests that incomplete pedigree information may led to biased estimates on variance components, consequently, on heritability. This effect was already reported by Junqueira et al. (2017). A similar result was observed by Aldridge et al. (2020) when evaluating several traits in swine. The goal with the use of metafounders is to make both pedigree and genomic information more compatible (Legarra, 2016; Meyer and Swan, 2019). In addition, van Grevenhof et al. (2019) argued that variance components from a model with metafounders might be more accurate after variance components rescaling, consequently the estimation of more accurate breeding values are expected. In fact, when pedigrees are well structured, the inclusion of genomic information might not cause an increase in heritability. However, the pedigree for the population used in this study has many individuals with unknown parents. The different results between genomic and non-genomic models come from a better estimation of relationships through SNP, when pedigree is incomplete. As observed by Junqueira et al. (2017), improved additive relationships can cause changes of additive and permanent environmental effects. In cases where a proper model is used and variance components are better estimated, higher heritabilities could be observed, which can benefit selection. This can help to boost the annual genetic gain in breeding programs because more reliable heritability estimate is translated into more accurate prediction of breeding values.

Genetic correlations (***r*_*a*_**) between Hereford and Braford were 0.67 (0.022), 0.45 (0.015), and 0.41 (0.017) for BLUP, ssGBLUP, and ssGBLUPm models, respectively. Note that under genomic models, the genetic correlation is lower than in BLUP. As stated by Hidalgo et al. (2020), variance components and genetic parameters based on **A** and **H** can be different if the population is under genomic selection. In such a case, genomic information is part of the selection process, and if the genomic information is not included, variance components can be biased. The genetic correlation is useful when designing breeding schemes and defining breeding objectives. In the case of genetic correlation between different breeds, our results show that some genomic regions responsible for the control of tick resistance are being expressed in both purebreds and crossbreds. This result indicates that the selection of Hereford for tick count resistance may also account for a positive impact on Braford resistance, when the latter originates from selected Hereford parents.

### Predictive Ability and Bias

**FIGURE 2:**
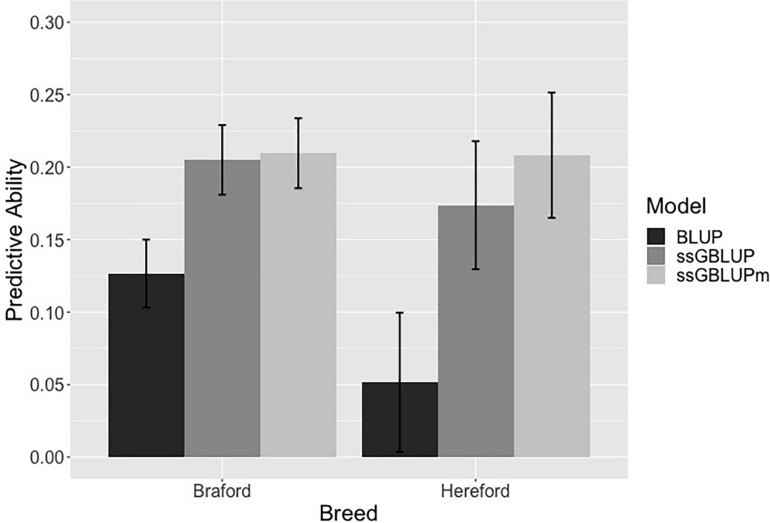
Predictive ability from a forward validation in Hereford and Braford when using pedigree (BLUP), single-step genomic BLUP (ssGBLUP), and ssGBLUP with metafounders (ssGBLUPm). Error bars represents the standard errors estimated using non-parametric bootstrapping.

The predictive ability for all 198 Hereford and 766 Braford animals used in the forward validation is in Figure 2 and Supplementary Figure S2. Forward validation is a good strategy to mimic the reality of breeding programs and genetics datasets, where breeding values of young animals are predicted based on data from older animals. As expected, the pedigree-based model had the worse predictive ability (0.051 and 0.126 for Hereford and Braford, respectively) when compared to ssGBLUP (0.173 and 0.205) and ssGBLUPm (0.208 and 0.209). With metafounders, there was an additional gain in predictivity for both breeds, especially for Herefords. This is because the number of phenotypes and genotypes available for Herefords is much smaller compared to Brafords, and any increase in prediction accuracy is expected to have a direct and positive impact under practical conditions when selecting breeding candidates. However, our results may still be limited by the size of the dataset, number of genotyped animals, and due to lack of animals with known parents in the pedigree. Perhaps, all allelic diversity present in the Hereford population could not be captured; therefore, further analyses using larger populations with more complete pedigree information are required to have a better understanding of the impact of using metafounders for the estimation of GEBV.

The degree of bias of the prediction methods is indicated by the coefficient of regression of phenotypes adjusted by fixed effects on (G)EBVs (Table 5). The optimal method to predict the genetic merit of animals would have a regression coefficient close to 1. For Hereford breed, the inclusion of metafounders provided the smallest bias and standard error. On the other hand, BLUP was the smallest biased model for Braford, with ssGBLUPm still showing the smallest standard error. According to Kennedy et al. (1988) relationships account for selection, drift, and non-random mating, but do not account for wrong definition of the base population or finite number of loci (Vitezica et al., 2011; Junqueira et al., 2017). Under those circumstances, fitting metafounders would contribute to the estimation of breeding values due to the addition of genetic relationships for founders of the populations. However, as the uncertainty of relationship increases, the variance of estimated breeding values may also increase. Consequently, the breeding values might show high bias, as it was observed on Braford ssGBLUP and ssGBLUPm. More studies are required to evaluate the benefits of the inclusion of metafounders under different proportions of known relationship information. In a simulation study, Bradford et al. (2019) observed the addition of metafounders led to less biased models, especially for traits with moderate to low heritability, as the case of tick count (*h* < 0.25).

**TABLE 5:** Regression coefficients (standard error) of phenotypes adjusted by fixed effects on (G)EBVs for young Hereford and Braford animals under pedigree and genomic models.

**Breed^*a*^**	**Model**	**Bias**
Braford	BLUP	1.11 (0.22)
	ssGBLUP	0.85 (0.10)
	ssGBLUPm	0.79 (0.09)
Hereford	BLUP	0.76 (0.46)
	ssGBLUP	1.28 (0.23)
	ssGBLUPm	0.89 (0.19)

*^*a*^BLUP, pedigree-based BLUP; ssGBLUP, single-step genomic BLUP; ssGBLUPm, ssGBLUP with metafounders.*

Our study shows the potential of the use of metafounders to increase the rate of genetic gain across generations due to a more acurate estimation of breeding values, in accordance to Xiang et al. (2017). Perhaps, the challenge for Brazilian breeding programs would be the availability of a large amount of marker information to calculate a more reliable and robust **Γ** since the matrix is built based solely on SNPs. This study focused on evaluating the impact of metafounders on the estimation of breeding values, with **Γ** being computed based on all genotyped animals. However, only a fraction (28%) of the population is genotyped and the number of genotyped animals is limited; which is the reality in almost all livestock populations. Therefore, there is still a lack of knowledge on how large the number of phenotyped and genotyped animals connected to metafounders should be needed to obtain accurate **Γ** estimates. Future studies should investigate the impact of the number of genotyped animals from different breeds on the estimates.

## Conclusion

The inclusion of genomic information in a multibreed Hereford/Braford population provides greater predictive ability than pedigree-based models for both breeds because of a better estimation of genetic relationships. When the level of pedigree missingness is high, the inclusion of metafounders can help to further increase the ability to predict future performance in small multibreed populations.

## Data Availability Statement

The datasets for this article are not publicly available because it is property of the Braford and Hereford producers, Embrapa, and GenSys Consultants and these information is commercially sensitive. For scientific research purposes, the data requests should be forwarded along with the research proposal to FC, fernando.cardoso@embrapa.br.

## Author Contributions

VJ executed the analysis and wrote the manuscript. PL, DL, FS, and FC provided support during analysis execution and reviewed the manuscript. All authors contributed to the article and approved the submitted version.

## Conflict of Interest

VJ was employed by the company Bayer and FC was employed by the company Embrapa Pecuária Sul. The remaining authors declare that the research was conducted in the absence of any commercial or financial relationships that could be construed as a potential conflict of interest.

## Footnotes

1 http://nce.ads.uga.edu/wiki/doku.php?id=documentation
