# Supplementary material for: Applying the Metafounders Approach for Genomic Evaluation in a Multibreed Beef Cattle Population
Source: Front Genet. 2020 Dec 3;11:556399. doi: 10.3389/fgene.2020.556399 (PMC7793833; doi:10.3389/fgene.2020.556399)
Supplement: Supplementary file 1 [file Data_Sheet_1.docx]

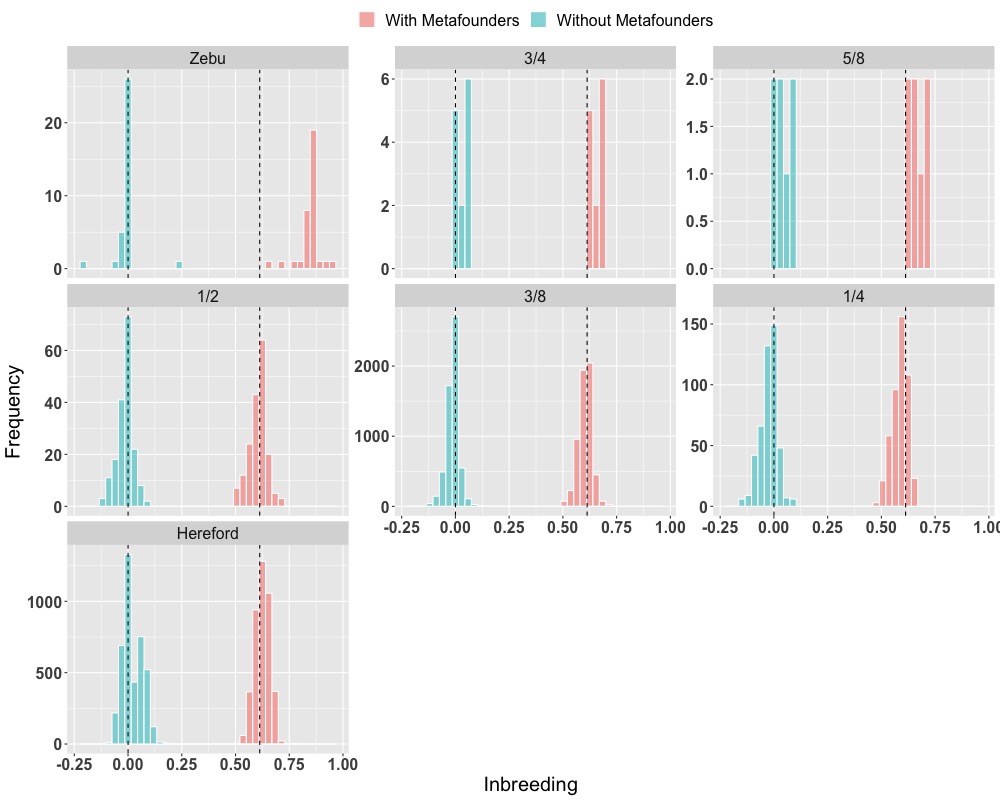


**Figure S1**. Inbreeding coefficients for Hereford, Zebu, and Braford animals. Coefficients were obtained from the diagonal elements of the realized relationship matrix (**H**) with (ssGBLUPm) and without metafounders (ssGBLUP). Dashed lines represent the average inbreeding from each relationship matrix.


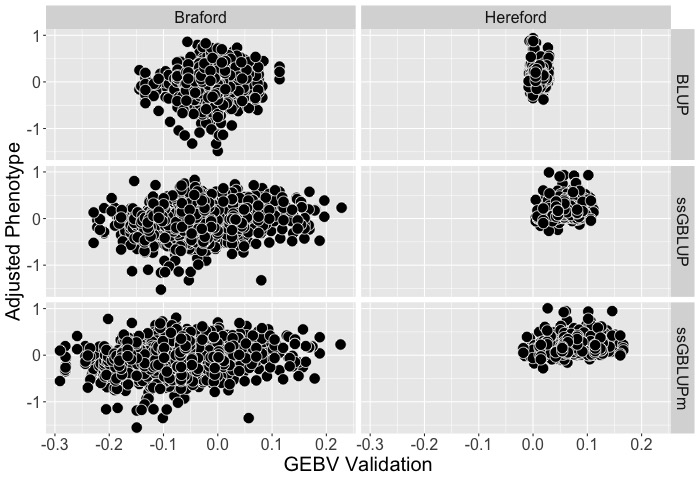


**Figure S2**. Association between phenotypes adjusted by fixed effects calculated using all data and breeding values (GEBV) estimated in the validation animals (198 Hereford and 766 Braford) using pedigree (BLUP), single-step genomic BLUP (ssGBLUP) , and ssGBLUP with metafounders (ssGBLUPm).
